# The power of many small sex differences in cognition, personality, and interests

**DOI:** 10.1038/s41598-026-53824-6

**Published:** 2026-05-27

**Authors:** Agneta Herlitz, Joakim K. E. Frostegård, Martin Asperholm, Richard Bränström, Elizabeth Guest, Joakim Martinsen, Hedda Sonnegård, Kimmo Sorjonen, Lisa B. Thorell, Björn N. Persson

**Affiliations:** Division of Psychology, Department of Clinical Neuroscience, Karolinska Institutet, Stockholm, Sweden

**Keywords:** Sex differences, Cognition, Personality, Interest, Prediction accuracy, Neuroscience, Psychology, Psychology

## Abstract

It has repeatedly been shown that most psychological sex differences are small, with largely overlapping distributions. This study examined whether multiple small sex differences—across cognitive performance, personality traits, and interests—can collectively distinguish between males and females and predict real-life outcomes. Participants (N_F_ = 1465, N_M_ = 1302, 35–45 years old) completed online tests and questionnaires that assessed cognitive performance, personality, and interest in people and in things. Results showed sex differences across 13 tasks and questionnaires in the expected directions. Importantly, when combined, these measures correctly predicted whether an individual was male or female in 80% of cases and accounted for a substantial share of the variance in the real-life outcome of occupational gender segregation. These findings demonstrate how multiple small individual differences can collectively yield substantial predictive power, offering new insight into the underpinnings of gender differences in society.

**Supplementary Information:**

The online version contains supplementary material available at 10.1038/s41598-026-53824-6.

## Introduction

Differences between men and women have long been a topic of great interest, regardless of whether they concern personality or cognition, are descriptive or inferential, or claim differences or no differences. Despite that, few, if any, researchers would argue that there is a “male” or “female” brain, or a clear dichotomy between male and female behaviors. Instead, it has repeatedly been shown that most psychological sex differences are small and have largely overlapping distributions^1–4^. Consequently, it has been suggested that men and women are more alike than different^5^, leading to the hypothesis that sex differences have minimal or no significant impact on individual lives or society at large^4,6^. While most psychological differences are small, in some real-life domains, such as occupational choices, differences are comparatively large^7^. In light of this, we aimed to investigate, first, whether variations in basic cognitive abilities, personality traits, and behaviors form a pattern that reliably distinguishes females from males, and second, whether these sex differences are meaningfully related to real-life outcomes, specifically occupational sex segregation (e.g.,^7–10^). We use the term “sex”, as it refers to the distinction (female/male) investigated in this study, although other non-binary conceptualizations of sex/gender exist. This choice of terminology is not intended to suggest that the observed differences are biologically rather than environmentally determined. Throughout the paper, we use “gender” for roles and identities, and in relation to concepts like equality, where “gender” is the commonly used term.

The presence of sex differences in some, but not all, cognitive functions is well documented. For example, females often outperform males in verbal tasks such as phonemic fluency (*d* ≈ 0.13^11^), verbal episodic memory, and face recognition (*d* ≈ 0.27^12,13^). Females also appear to hold an advantage in emotion recognition, though the size of this effect varies considerably across studies, ranging from nearly negligible (*d* ≈ 0.07^14^), to small (*d* = 0.19^15^), to medium-sized (*d* = 0.47^16^). Males, on the other hand, perform better on spatial tasks, particularly on those requiring mental rotation, but also on those assessing spatial perception, such as line matching (*d* ≈ 0.48^17^). While cognitive sex differences are often modest in magnitude^18^ and poorly understood^19^, they have been consistently reported throughout the lifespan and across geographical regions^12,13,17,20–23^.

There are also reliable sex differences in personality traits as measured in the five-factor model of personality^20–22^, with females typically scoring higher on extraversion (*d* = 0.15^23^), agreeableness (*d* = 0.56^23^), and neuroticism (*d* = 0.41^23^), but also on the traits conscientiousness (*d* = 0.12^22^) and openness (*d* = 0.19^22^). Some of these differences are small, likely because there are sex differences in different directions at the more specific facet level (e.g.,^22^). For instance, whereas females tend to score higher on warmth and positive emotions, males score higher on assertiveness and excitement seeking, making the sex difference in extraversion small^24^. Most of these sex differences have been reported across geographical regions^23,25^.

Within personnel selection research, the orientation of interests toward the social environment (“people”) or the physical environment (“things”) has often been conceptualized as two opposing poles of a single dimension^26,27^. This framework consistently reveals a large sex difference (*d* ≈ 0.93^28^) observed across multiple countries^23^. However, the univariate conceptualization of the people-things orientation has been questioned, with critics arguing that strong interest in people does not necessarily equate a weak interest in things, and vice versa. In response to this critique, a bivariate instrument was developed^29^, focusing on everyday interests rather than vocational preferences, such as interest in listening in on a conversation between two people in a crowd, or stopping to watch a machine working on the street. Using this instrument, Woodcock and colleagues found that females, compared to males, expressed greater interest in people (*d* = 0.49), while males showed greater interest in things (*d* = 0.99^30^).

Although the interpretation of effect sizes, such as classifying them as small, medium, or large, is context dependent and their practical significance is debated^31^, most psychological sex differences are considered small, with effect sizes (*d*s) typically ranging between 0.2 and 0.5. Medium- (*d* = 0.5—0.8) or large-sized effects, such as those observed in interest^30^, are relatively rare^2,4^. Whether these differences carry any significance has been subject to much debate in psychology, with some arguing that men and women are mostly similar across traits and that differences are overstated^2,5^. Others have shown that while individual personality traits typically show substantial overlap between male and female distributions, this overlap shrinks markedly once multiple traits are considered jointly^25,32,33^. Determining what constitutes a meaningful difference is not straightforward, but one approach to evaluating the meaningfulness of a difference is to assess whether it can reliably predict category membership.

To explore this, we investigated whether an individual’s sex could be predicted from a combination of their cognitive performance, personality traits, and interests. The predictors were selected to encompass a wide range of well-studied, basic psychological phenomena that consistently show mostly small sex differences across cultures and throughout the lifespan. We avoided tasks and questionnaires that are strongly and directly tied to sex, such as gender expression or what sex one is attracted to^34^, as we thought that their inclusion would trivialize prediction and thus render results uninteresting. Altogether 2,767 individuals, aged 35 to 45 years, completed 13 tasks and questionnaires assessing episodic memory, verbal fluency, spatial abilities, and emotion recognition, and additionally provided self-reports on personality as well as interests in people versus things. They also reported their current occupation, for which sex distribution scores were derived from national registry data. We hypothesized that, when considered jointly, many small psychological sex differences would form a coherent pattern clearly distinguishing between the sexes. Furthermore, we aimed to examine whether the combined differences would predict the real-life outcome of gender segregation in occupational choice, thereby shedding light on how multiple small sex differences are associated with differences in life choices.

## Method

### Participants

Participants were recruited through advertisements on social media sites (Facebook and Instagram) as well as on Karolinska Institutet’s webpage between February and May, 2024. After approximately two weeks, the advertisements were reconfigured to only be shown to men to achieve a more even sex ratio. A website gave information about the study and contained a link to a digital form in which individuals could express interest in participating by submitting their year of birth and email address. People meeting the age criteria for inclusion (age 35 to 45 years) were sent an email with a link to the study web application and a unique login code. The narrow age range was chosen to reduce age-related variation and target individuals with established careers.

By May 5th, 2024, 3096 individuals had logged in and confirmed informed consent. Out of these, 236 participants immediately left the website and were thus excluded. In addition, participants who indicated that their birth sex did not correspond to their legal sex were excluded (*n* = 20). We excluded participants who reported having transitioned or being under transition, as it would involve taking sex contrary hormones, which may influence cognitive performance and behavior. Participants were also excluded based on having insufficient knowledge of Swedish (*n* = 6), falling outside of the age range (*n* = 18), reporting an invalid occupation (*n* = 2), or because of missing data in all study tasks (*n* = 47). Furthermore, we excluded individual observations that, for instance, exceeded time limits between episodic memory encoding and retrieval (details about exclusion criteria are available here: https://osf.io/kfxp3). In total, 329 participants were excluded, rendering a final sample of 2767 participants (*n*_M_ = 1,302, *n*_F_ = 1465).

Compared to females, males were slightly but significantly older (*M*_M_ = 40.8, *SD*_M_ = 3.1; *M*_F_ = 40.4, *SD*_F_ = 3.1; *t* = 3.12, *p* = 0.002), less likely to have post-high-school education, and had fewer years of post-high-school education. More specifically, 89% of females and 74% of males reported having three or more years of higher education which is higher than in the general population, in which 61% of females and 45% of males have some post-high-school education^35^.

Ethical approval was granted by the Ethical Review Authority before study commencement (ID: 2023-06414-01 and 2023-06414-02). All participants provided informed consent prior to participation. All methods were performed in accordance with the guidelines and regulations of the Swedish Ethical Review Authority. No compensation for participation was provided, but the participants had the option to receive a summary of their test results upon completion.

### Procedure

The study web application was developed using jsPsych v6^36^. Participants could participate using a computer (not a phone or tablet) and were asked to sit in a quiet room where they would not be disturbed. After the mandatory informed consent form, the web application automatically entered full screen mode and basic *demographic information* was collected (tasks and questionnaires in *italics* are used in the current study). Thereafter, the tasks were administered in the following order: (1) *encoding of a verbal episodic memory task*, (2) *line angle judgment* test; (3) *free recall of the verbal episodic memory task*; (4) *people-things interest*; (5) questionnaire on educational achievement; (6) *questionnaire on current occupation*; (7) *verbal episodic memory recognition task*; (8) *encoding of faces for the episodic face recognition* task, (9) *verbal fluency task*; 10) *face recognition task;* (11) political view; (12) *mental rotation*; (13) *personality assessment*; (14) *emotion recognition*; (15) gender expression information; (16) typing skills; (17) information on mental health. All tasks and questionnaires were in Swedish. Participants were able to return to the test battery between tasks, and there was no total time limit. As a result, some participants left the survey open on their computer and returned to it later. The median response time was 42 min. After completing all tasks, participants were able to view summaries of their performance in the cognitive tasks, as well as summaries of their answers pertaining to political views and personality.

## Material

### Background information

Participants were requested to provide information regarding their date of birth (year and month), current legal sex, legal sex at birth as well as their level of Swedish proficiency (rated on a 5-point Likert-scale ranging from 1 (novice) to 5 (fluent). Participants were also asked whether they had completed primary school (grades 1–9) or an equivalent education abroad and whether they had passed upper secondary school (or equivalent abroad). Additionally, they provided information on higher education, current employment status, and what occupation they wished they had.

### Verbal episodic memory

To assess verbal episodic memory, participants were presented with a sequence of 28 words, presented one word at a time for 2.5 s followed by a 0.5 s pause before the next word was shown. After a brief interval (approximately 1.5 min), during which participants responded to the line angle task (see below), a free recall task commenced in which participants were asked to write down as many remembered words as possible. The outcome variable was the number of accurately recalled words. Approximately 6 min later, the participants were presented with 56 words, 28 of which were displayed during encoding, and were instructed to indicate in a “yes”/”no” recognition task whether they recognized the word or not. The verbal episodic outcome variable was a composite value formed by z-transforming the number of correct words recalled and recognized (hits minus false alarms), which were combined and transformed into a z-score. Although the wordlist to-be-remembered in this task was developed specifically for this battery of tasks, similar tasks have previously been shown to yield sex differences favoring females^12^.

### Face recognition

To assess the ability to remember and recognize faces (episodic memory), participants were presented with 20 colored photographs of faces with their hair hidden (23) and were told to try to remember these^37^. Each of the 20 target faces was shown for 3 s. Commencing recognition, approximately 3.5 min later, participants were asked to identify in a “yes”/”no” recognition task whether each of the 40 images, 20 “old” and 20 “new”, had been shown during encoding. The outcome variable was correctly recognized faces (hits minus false alarms), with the maximum score being 20. This task has previously been shown to yield sex differences favoring females^12^.

### Line angle judgment

To evaluate two-dimensional spatial ability, participants were presented with a reference line at an angle, and beneath it, 15 lines that varied between − 90 degrees and + 90 degrees relative to the reference line. Participants were asked to select the line that matched the angle of the reference line. There were two practice trials and 20 test trials, each with a 5 s time constraint after which the reference-line disappeared, but participants could still respond. The outcome variable was the number of correct responses, with the maximum score being 20. Cronbach’s alpha for this task was 0.80. The task was adapted from^17^ and is known to reveal sex differences, with males generally outperforming females.

### Mental rotation

To assess three-dimensional spatial ability, a revised version of the Mental Rotation Task^38^ was used. Participants were shown a reference 3D model at the top and another set of four 3D models below it. Using checkboxes, participants selected the two models from the bottom set that depicted the same object as the reference model at the top, albeit rotated in three-dimensional space. Following practice trials, participants were presented with 10 trials in which the reference model was shown for 16 s before disappearing, after which it was still possible to answer. The outcome variable was the total number of correct identifications subtracted by any incorrect selections, with maximum score being 20. Cronbach’s alpha was 0.66. This task is known to typically reveal sex differences, with males outperforming females^39^.

### Verbal fluency

To assess verbal production, participants were asked, in three different trials spanning one minute, to write down as many words as they could beginning with the letter F, A and S, respectively. Automated scoring was implemented using a dictionary, meaning that misspellings were rejected. The outcome variable was a composite value formed by z-transforming the number of correct words generated for F, A, and S, which were combined and transformed into a z-score. Cronbach’s alpha for verbal fluency was 0.87. This task is known to typically show sex differences, with females outperforming males^11^.

### Emotion recognition

To evaluate emotion recognition, a revised version of the Reading the Mind in the Eyes test was employed^40,41^. Participants were presented with a gray-scale image displaying the eye region of an individual, accompanied by the prompt ‘What emotion does the person express?’ and four response choices, varying across trials. No time constraints were imposed. After one practice trial, 36 test trials were administered and the outcome variable was the number of correctly identified emotions. Cronbach’s alpha was low, at 0.54. This test has been shown to yield sex differences favoring females^42^.

### Personality

To assess personality, we used the Swedish version^43^ of the Big Five Inventory-2^44^ which consists of 60 items aimed at measuring personality from 5 personality dimensions: Extraversion, Agreeableness, Conscientiousness, Neuroticism, and Openness. The dimensions are constructed by computing a mean for 12 items for each dimension. Items consist of short statements rated on a 5-point Likert-scale ranging from 1 (strongly disagree) to 5 (strongly agree). Item scores were reversed for 30 items in accordance with scoring instructions. Cronbach’s alphas were: Extraversion (0.85), Agreeableness (0.82), Conscientiousness (0.85), Neuroticism (0.89) and Openness (0.82).

### People-things interest

To assess interest in people and things, a short and adapted version of the Person–Thing Orientation Scale^29^ was used. It contained 13 items assessing how participants orient themselves towards different aspects of their environment. Participants were asked to rate on a 5-point Likert-scale how much they would enjoy certain situations even if they had never experienced them (1 = “not at all”, 2 = “a little”, 3 = “moderately”, 4 = “quite a bit”, and 5 = “very much”). Examples of situations were “listening in on a conversation between two people in a crowd” (people-oriented) and “trying to fix your own toaster” (things-oriented). Cronbach’s alphas were 0.85 and 0.75 for interest in things and people, respectively. This measure refers to more general interests and should not be conflated with more specific, work-related, interests such as those in the RIASEC model (cf.^29^).

### Occupation

Participants were provided with a list of 429 occupational categories^45^ and were asked to select the one most accurately describing their current or most recent occupation. In addition to the titles of the occupational categories, descriptions were displayed. Sex distribution within each occupational category, ranging from 0 (only males) to 1 (only females), was calculated from available registry data^46^, based on 35- to 44-year-olds living in Sweden. For example, a participant selecting “preschool teacher” would receive a score reflecting the national sex distribution for that occupation (i.e., 0.96) among 35- to 44-year-olds. Out of 429 possible occupations, our sample held 336 unique occupations. A total of 38 participants provided no response to this question. Additionally, 628 participants gave a response but reported not finding a suitable occupation in the list. This set of responses was independently recoded by two research assistants, and based on the participants’ description of their occupation, 103 out of 628 participants were given new codes. Among 182 participants who reported being students, 104 were recoded to the occupation they were studying for. Interrater agreement was high or relatively high for both recoding procedures (Cohen’s κ = 0.89 and κ = 0.69, respectively).

### Statistical analyses

All task scores were transformed into z-scores using the mean and standard deviation across all individuals. The study’s main analyses used binomial logistic regression with sex as the outcome variable. Four nested models were tested to assess the predictive value of each broad class of variables. Model 1 included cognitive variables for which previous research has shown a female advantage (i.e., verbal fluency, verbal episodic memory, face recognition and emotion recognition). Cognitive variables with a male advantage (i.e., angle judgment and mental rotation) were then added in Model 2. Personality (i.e., openness, conscientiousness, extraversion, agreeableness, neuroticism) was added to Model 3 and, finally, interest in people and in things in Model 4. These models were based on complete data in order to facilitate model fit comparisons. There were 2,474 complete observations across the variables entered in the final regression models (13 z-scores + outcomes). In addition to logistic regression, beta regression was used to investigate the contribution of the 13 cognitive, personality, and interest variables to predict the sex distribution in the participants’ occupational choice. Beta regression is appropriate when the outcome variable is bounded between 0 and 1, which is the case for our occupational segregation variable, which is a proportion. Three beta regression models were used. The first included only sex as a covariate, the second included the 13 variables without controlling for sex, while the third included sex as well as the 13 variables.

Logistic regression models were evaluated both in terms of their predictive accuracy and with various model metrics, such as the Akaike Information Criterion (AIC), Bayesian Information Criterion (BIC), Root Mean Squared Error (RMSE), χ2, Tjur’s *R*^2^ and Nagelkerke’s *R*^2^. Lower values for AIC, BIC, and RMSE and higher *R*^2^s indicate better fit between the observed data and model predictions. Tjur’s *R*^2^ reflects how well a model discriminates between cases, where 1 indicates perfect discrimination and 0 no discriminative power. Nagelkerke’s *R*^2^ reflects improvement in the log-likelihood over the null model, and a better model has a value closer to 1. Neither of these statistics are meant to be interpreted in terms of explained variance but reflect model fit and average discrimination power and are particularly informative when comparing models. We attach no particular value to the order in which the variables were entered in the analyses, although the variables are grouped thematically (e.g., female favoring cognitive tasks, male favoring cognitive tasks, personality, interest). Nevertheless, to address potential concerns about predictor ordering, we conducted dominance analysis^47^, which evaluates the relative importance of each predictor by comparing its incremental contribution to model fit across all possible sub-model combinations, yielding results that are invariant to entry order. We also conducted tenfold cross-validation to assess the generalizability of the final model. Both analyses are reported as supplementary information and support the robustness of the reported results.

## Results

### Sex differences

As shown in Table 1, there were small to large differences between males and females in the expected directions. Females performed at a higher level than males on cognitive tasks assessing verbal fluency, verbal episodic memory, face recognition, and emotion recognition. In contrast, males outperformed females on the two spatial tasks, line angle judgment and mental rotation. Additionally, females scored higher on all personality dimensions, with the difference in openness being the smallest. There were also significant sex differences in interests, with males showing greater interest in things and females showing greater interest in people.

**Table 1:** Descriptive statistics (Z-scores) for each sex, Cohen’s d and variance ratios (VR).

Predictor	Males			Females			Group Comparison	
	*N*	*M*	*SD*	*N*	*M*	*SD*	Cohen’s *d* (95% CI)	VR
Verbal Fluency	1254	− 0.17	0.98	1427	0.15	0.99	0.32 (0.24, 0.40)	0.99
Verbal Episodic Memory	1239	− 0.23	0.98	1399	0.20	0.97	0.44 (0.36, 0.52)	1.02
Face Recognition	1234	− 0.16	0.98	1415	0.14	1.00	0.30 (0.22, 0.37)	0.95
Emotion Recognition	1210	− 0.11	1.02	1394	0.09	0.98	0.20 (0.13, 0.28)	1.08
Line Angle Judgment	1298	0.35	1.00	1456	− 0.31	0.90	− 0.70 (− 0.77, − 0.62)	1.23
Mental Rotation	1216	0.36	0.91	1396	− 0.31	0.97	− 0.72 (− 0.80, − 0.64)	0.88
Openness	1216	− 0.06	1.01	1394	0.05	0.99	0.10 (0.03, 0.18)	1.03
Conscientiousness	1216	− 0.23	1.00	1394	0.20	0.96	0.44 (0.36, 0.51)	1.08
Extraversion	1216	− 0.17	1.00	1394	0.15	0.97	0.32 (0.24, 0.40)	1.06
Agreeableness	1216	− 0.19	1.05	1394	0.16	0.93	0.35 (0.27, 0.43)	1.29
Neuroticism	1216	− 0.23	0.99	1394	0.20	0.96	0.44 (0.36, 0.52)	1.05
Interest in People	1287	− 0.19	1.00	1454	0.17	0.97	0.37 (0.30, 0.45)	1.06
Interest in Things	1287	0.51	0.91	1454	− 0.45	0.84	− 1.11 (− 1.19, − 1.02)	1.17

Total *N* varies between 2604 and 2754. Cohen’s *d* is reported with 95% confidence intervals in parentheses. VR = variance ratio (*SD*^2^_men_ / *SD*^2^_women_); values above 1.0 indicate greater male variability.

### Predicting sex

In the regression analyses, we investigated if we could predict the sex of the participants based on their performance on the completed tasks. The binomial logistic regressions included four steps, added consecutively. As shown in Table 2, each added step significantly contributed to prediction accuracy. Sensitivity (true positives /(true positives + false negatives)), specificity (true negatives / (true negatives + false positives)) and accuracy (correct predictions / total predictions) also increased substantially with each step (cf. Table 3). Multicollinearity was not an issue, with all VIF’s well below 4 (range 1.04–1.37). Each consecutive model showed better fit than the previous one, as assessed by *AIC, BIC, RMSE* and pseudo-*R*^2^. More specifically, the decrease in *RMSE* is indicative of improvement in the predicted probabilities for each model. *AIC* and *BIC* use the log-likelihood and penalize model complexity to adjudicate between comparable models. In this case, each subsequent step showed better fit despite having more parameters.

**Table 2:** Regression coefficients with 95% confidence intervals predicting female sex.

Predictors	Model 1		Model 2		Model 3		Model 4	
	OR	95% CI	OR	95% CI	OR	95% CI	OR	95% CI
(Intercept)	1.15	1.06–1.25	1.20	1.09–1.31	1.21	1.10–1.34	1.21	1.09–1.35
Verbal Fluency	1.26	1.16–1.37	1.48	1.34–1.63	1.43	1.29–1.59	1.31	1.17–1.46
Verbal Episodic Memory	1.43	1.31–1.56	1.57	1.43–1.74	1.61	1.45–1.80	1.74	1.55–1.95
Face Recognition	1.23	1.13–1.34	1.26	1.14–1.39	1.27	1.14–1.41	1.20	1.08–1.35
Emotion Recognition	1.09	1.00–1.18	1.16	1.05–1.28	1.14	1.03–1.27	1.13	1.01–1.26
Line Angle Judgment			0.48	0.43–0.53	0.49	0.44–0.55	0.50	0.44–0.56
Mental Rotation			0.42	0.38–0.47	0.47	0.42–0.52	0.56	0.50–0.64
Openness					0.85	0.76–0.95	0.95	0.84–1.06
Conscientiousness					1.65	1.47–1.84	1.58	1.41–1.78
Extraversion					1.28	1.15–1.43	1.25	1.11–1.41
Agreeableness					1.49	1.34–1.67	1.33	1.17–1.51
Neuroticism					2.23	1.99–2.52	1.93	1.71–2.20
Interest in People							1.02	0.90–1.16
Interest in Things							0.41	0.36–0.46
*AIC*	3254.9		2655.0		2383.0		2162.2	
*BIC*	3284.0		2695.7		2452.7		2243.5	
*RMSE*	0.48		0.42		0.40		0.37	
*df*	5		7		12		14	
Δχ^2^	172.5		603.9		282.1		224.8	
Tjur’s R2	0.07		0.28		0.37		0.44	
Nagelkerke’s R2	0.09		0.36		0.46		0.54	

Females are coded 1 and males 0. *AIC* = Akaike Information Criterion, *BIC* = Bayesian Information Criterion, *RMSE* = Root Mean Square Error. Δχ^2^ reflects the likelihood ratio test comparing each model to the preceding model.

**Table 3:** Confusion matrices from each logistic regression model.

Model	Observed			Accuracy		
	Prediction	Male	Female	Male	Female	Overall
Model 1: Verbal-Episodic	Male	582	395	0.51	0.70	0.61
	Female	568	929			
Model 2: Verbal-Episodic + Spatial	Male	789	314	0.69	0.76	0.73
	Female	361	1010			
Model 3: Verbal-Episodic + Spatial + Personality	Male	847	277	0.74	0.79	0.77
	Female	303	1047			
Model 4: Verbal-Episodic + Spatial + Personality + Interests	Male	886	234	0.77	0.82	0.80
	Female	264	1090			

The cells with predictions list predicted values (where values above 0.5 are classified as ones and below 0.5 are classified as zeroes) and compare these with observed values. The diagonal represents correct predictions and the off-diagonal incorrect predictions.

**Fig. 1:**
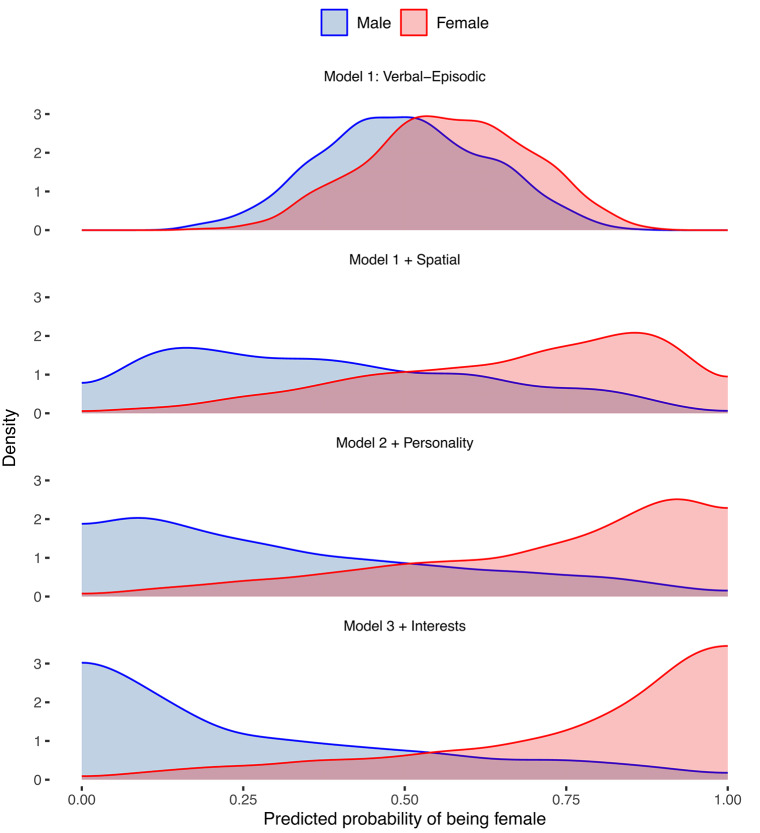
Predicted probabilities from logistic regression models step 1 to 4.

In the final model (see Table 2), the best predictors of sex were mental rotation (OR = 0.56, *z* = −9.2, *p* < 0.001), verbal episodic memory (OR = 1.74, *z* = 9.5, *p* < 0.001), neuroticism (OR = 1.93, *z* = 10.2, *p* < 0.001), line angle judgment (OR = 0.50. *z* = −12.0, *p* < 0.001), and interest in things (OR = 0.41, *z* = −14.1, *p* < 0.001). Thus, for each one standard deviation-unit increase in interest in things, the odds of being female decreases by 59%, holding the other modelled variables constant. This further suggests that, on average, a higher score on interest in things is associated with a lower likelihood of being female. The incremental improvement in prediction is shown graphically in Fig. 1. More information from simple logistic regression models is reported as SI.

In Table 3, results from confusion matrices from each model are presented. A confusion matrix consists of a 2 × 2 table with observed (ground truth) and predicted (model estimated) values. This table displays an overview of model estimations of true positives and negatives (correct predictions) and false positives and negatives (incorrect predictions). As sensitivity and specificity are ambiguous when it comes to prediction of sex, we use “accuracy males” (sensitivity) and “accuracy females” (specificity) as clarifying terminology (cf. Table 3).

**Fig. 2:**
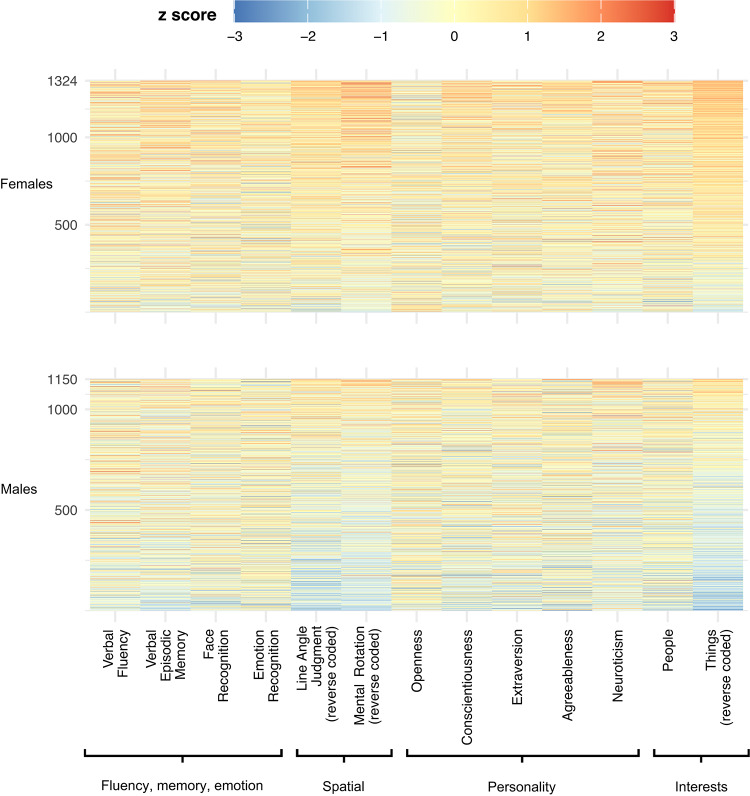
Heatmap showing the degree of “maleness” or “femaleness” reflected in each task or questionnaire is shown for female participants (top) and male participants (bottom). Each horizontal line represents an individual participant, while each column corresponds to a specific task or questionnaire (x-axis). Shades closer to red indicate a more “feminine” performance or response, whereas shades closer to blue indicate a more “masculine” performance or response. Approximately 0.25% of z-scores that were smaller or larger than ±3 are displayed as ±3 (see SI for more details).

In the final model, 886 (77%) of the males and 1090 (82%) of the females, were correctly classified. Thus, the overall model accuracy was 80%. McNemar’s Test was insignificant (*p* = 0.19) which indicates that the model does not appear biased in favor of predicting one sex more accurately than the other. In Fig. 1, each model’s predicted probabilities are also plotted to better visualize the incremental improvement in prediction accuracy. Figure 2 displays a heatmap showing the degree of “maleness” or “femaleness” reflected in each task.

As a post-hoc test of model robustness, we excluded the three best predictors (neuroticism, line angle judgment, and interest in things) and ran a new logistic regression. This new model resulted in an overall accuracy of 0.70, making correct predictions for 65% of males 74% of females. The best predictors were mental rotation (OR = 0.42, *z* = 15.9, *p* < 0.001) and verbal-episodic memory (OR = 1.52, *z* = 8.5, *p* < 0.001). This suggests that the models were relatively robust to removal of even the largest predictors, and that our results are not driven by single variables.

### Predicting occupation segregation

Finally, to provide context and relevance for our findings, we used beta regression, suitable for ratio outcomes, with gender segregation in participants’ occupation as outcome, operationalized as the population-wise proportion of people with that occupation being women. We used three models, one only including sex as a covariate, one including the 13 tasks used in Model 4 previously, and a final model including both sex and the 13 tasks. We elected to calculate average marginal effects (AMEs) for ease of interpretation and present coefficients from the final model below and in Table 4 (see SI for additional information). In terms of explained variance, pseudo-*R*^2^ for just sex was 0.126, while it was 0.204 for the 13 tasks. In the final model with tasks and controlling for sex, the pseudo-*R*^2^ was 0.220. The largest effect was found for the control variable sex, with an average slope of 0.083, which means females work in occupations that have 8.3 percentage points more females than the occupations males work in. Among the other covariates, interest in things was again the strongest predictor, with an average slope of −0.072, indicating that a 1 SD increase in interest in things is associated with a 7.2 percentage point decrease in the proportion of females in one’s occupation. The other significant (*p* < 0.01) predictors were interest in people (AME = 0.023), extraversion (AME = −0.017), and mental rotation (AME = −0.016). This implies that when comparing a male who is 1 SD above the mean in interest in things with a female who is 1 SD below the mean in interest in things, the male would be predicted to work in occupations with approximately 22.7 percentage points fewer females.

**Table 4:** Regression results using gender segregation in occupation as criterion.

Predictor	Estimate	*SE*	*p*
Sex	0.083	0.011	< 0.01
Verbal Fluency	0.003	0.004	0.50
Verbal Episodic Memory	0.004	0.004	0.33
Face Recognition	0.000	0.004	0.93
Emotion Recognition	− 0.002	0.004	0.73
Line Angle Judgment	− 0.008	0.004	0.08
Mental Rotation	− 0.016	0.005	0.00
Openness	0.006	0.005	0.19
Conscientiousness	− 0.001	0.005	0.79
Extraversion	− 0.017	0.005	< 0.01
Agreeableness	0.004	0.005	0.47
Neuroticism	0.000	0.005	0.96
Interest in People	0.023	0.005	< 0.01
Interest in Things	− 0.072	0.005	< 0.01

Estimates are average slopes.

To clarify how much of the sex gap in occupational gender segregation is associated with performance on tested psychological domains versus non-tested effects of sex, we generated predictions for four groups: men and women crossed with male-typical and female-typical cognitive, personality, and interest profiles. These profiles were derived empirically, with each representing the observed mean values across all measured dimensions within the respective sex (male/female), enabling direct comparison between performance configurations. Men with a male-typical profile were predicted to work in occupations that are 43.4% female, whereas men with a female-typical profile were predicted to work in occupations that are 53.1% female, a 9.7 percentage point difference attributable to the performance profile alone. The corresponding figures for women were 52.0% and 61.5%, yielding a similar 9.5 percentage point performance effect. The direct effect of sex was similarly consistent: holding a male-typical profile constant, women were predicted to work in occupations with 8.6 percentage points more females than men (52.0% vs. 43.4%); holding a female-typical profile constant, the difference was 8.4 percentage points (61.5% vs. 53.1%). Thus, the effect of performance differences was comparable in magnitude to the non-tested effect of sex. Comparing women with a female-typical profile to men with a male-typical profile, the total sex gap was 18.1 percentage points, reflecting the joint association of psychological performance differences and non-tested sex-related factors with occupational gender segregation.

## Discussion

We aimed to determine whether it is possible to predict an individual’s sex based on their cognition, personality, and interests. Specifically, we wanted to know if the potential additive impact of several sex differences, most of them small, would result in a non-negligible separation between males and females. Our selection of 13 tasks and questionnaires was based on the following criteria: they needed to assess psychological dimensions showing sex differences across the lifespan and across diverse cultural contexts while not being trivially linked to sex (e.g., sexual attraction). We found reliable sex differences in all tested domains, with the majority being small (*d* = 0.10–0.44), two of medium magnitude (*d* ≈ 0.70; spatial abilities), and one large effect size (*d* = 1.11; interest in things). When combined, cognitive performance, personality, and interests could correctly predict whether an individual is male or female in 80% of cases. Furthermore, these sex differences could also reliably predict the real-life outcome of gender segregation in the participants’ occupations, explaining 22% of the variance, albeit with substantial shared variance between sex and the tasks. Additionally, our analyses showed a larger contribution from the performance on the tested domains (~ 9.7%) than from non-tested sex factors (~ 8.5%) on occupational sex segregation. Taken together, the results demonstrate that differences in basic psychological domains, when considered jointly, allow relatively accurate predictions of sex and of gender segregation in the participants’ occupations, thereby contributing to an initial understanding of how many small differences are associated with larger sex differences in life choices.

Can an 80% classification accuracy in predicting an individual’s sex be considered high? For context, structural MRI scans—when controlling for head size—achieve about 60% accuracy^48^. When head size is included, accuracy increases to between 69 and 93%^49–51^. This suggests that our result is relatively strong. Importantly, even after excluding the three tasks with the largest observed sex differences (i.e., interest in things, mental rotation, and line angle judgment), the model maintained a relatively high classification accuracy of 71%. This underscores the notion that many small differences can collectively create a large effect.

Could the accuracy be improved further? Yes. For instance, predictors that are strongly and directly tied to sex, such as sexual attraction (i.e., the sex one is attracted to^34^) or body morphology^52^, could substantially improve predictive accuracy. In addition, incorporating psychological variables with well-documented, albeit small, sex differences—such as tendencies toward positive and negative emotions^53^—would likely further enhance predictive power. Accuracy could also be increased by using more fine-grained predictors, for instance, individual test items rather than domain-level composites^25,32,33^, or by applying machine learning methods capable of capturing complex interactions among variables. However, the primary aim of this study was not to maximize prediction accuracy, but to demonstrate that a clear cumulative pattern emerges from many small differences in broad, well-established psychological dimensions, which led us to use domain-level scores corresponding to psychologically interpretable constructs (e.g., spatial ability, agreeableness, and interest in things).

While prior research has shown that the overlap between male and female distributions decreases substantially when multiple traits are considered jointly^25,32,33^, we extend this work by demonstrating that several small differences across a range of psychological dimensions can accumulate into substantial disparities, which in turn are associated with real-life outcomes, such as occupational choice. One important implication of the results is that even small sex differences may be meaningful, particularly when considered in combination. Human behavior is rarely determined by single traits but shaped by complex constellations of abilities, preferences, and personality characteristics. Consistent with this, our results showed that multiple psychological variables exhibiting sex differences meaningfully predicted the real-world outcome of gender segregation in individuals’ occupational choices. This pattern suggests that multivariate psychological profiles may be associated with the horizontal gender segregation observed in educational and career pathways, thereby furthering our understanding of the psychological correlates of sex segregation in life choices. At the same time, sex remained a significant predictor of occupational outcomes after accounting for cognitive performance, personality, and interests. This is consistent with the view that structural factors may also be associated with segregation independently of psychological sex differences^54^.

## Strengths, limitations, and future directions

While this study benefits from a large sample, it also has certain limitations. First, the lack of control over the online test environment could impact the consistency of results. However, since this limitation affects both male and female participants equally, we do not expect it to have influenced the overall pattern of findings. Furthermore, we cannot conclude that the participants’ cognitive abilities, personality, and interests cause their choice of occupation, merely describe the associations between them. Although there is a risk of reversed causation, it should be noted that sex differences in cognition, personality, and interests are reported throughout the life-span, which makes this alternative less likely^12,13,53,55^. Finally, the outcome variable “sex” was operationalized as the correspondence between legal sex at birth and current legal sex. We did not analyze differences based on gender identity or adopt a more nuanced non-binary perspective on gender. This decision was informed by both the binary framework employed in prior studies upon which our research is built, and practical considerations related to sample size. In line with findings from prior studies^56^, we found that the proportion of individuals who report a gender identity other than their sex assigned at birth is very small (0.5–0.6%). Future research using sufficiently large samples should employ a wider perspective on sex and gender and include individuals with a different gender identity than the gender they were assigned at birth.

## Conclusions

Our results support previous findings, confirming sex differences in cognitive performance, personality, and interest in people and things. More importantly, our results illustrate that the differences in these domains, when considered together, allow rather accurate predictions of sex. While determining how well several small differences in cognition and behavior can differentiate males from females may be of substantial theoretical interest, the more important implication of this research lies in its potential to shed light on real-world outcomes. Specifically, we found that these psychological characteristics were meaningfully associated with the degree of gender segregation in the individual’s occupational choices. These findings suggest that even subtle sex differences, when aggregated, may contribute to the persistence of gendered patterns in behavior and life trajectories.

## Supplementary Information

Below is the link to the electronic supplementary material.

Supplementary Material 1

## Data Availability

Study materials, data, and analysis scripts are publicly available (https:/osf.io/kfxp3). All analyses can be reproduced with the shared data with the caveat that one participant was excluded from the shared data for identifiability reasons.
