# Supplementary material for: The power of many small sex differences in cognition, personality, and interests
Source: Sci Rep. 2026 May 27;16:16572. doi: 10.1038/s41598-026-53824-6 (PMC13219607; doi:10.1038/s41598-026-53824-6)
Supplement: Supplementary file 1 — Supplementary Material 1 [file 41598_2026_53824_MOESM1_ESM.docx]

**Supplementary Information**

**The power of many small sex differences in cognition, personality, and interests**

Agneta Herlitz

Joakim K. E. Frostegård

Martin Asperholm

Richard Bränström

Elizabeth Guest

Joakim Martinsen

Hedda Sonnegård

Kimmo Sorjonen

Lisa B. Thorell

Björn N. Persson

Division of Psychology,

Department of Clinical Neuroscience,

Karolinska Institutet, Stockholm, Sweden

# Descriptive statistics

In the main manuscript, we report descriptive statistics divided by sex. Descriptive statistics for the full sample are reported in Table SI1, below. In both the main manuscript and SI, we use x-axis ranges of 3 standard deviations for plots, but not all values fall inside that range, as indicated by minimum and maximum values > |3|. In total, 76 values out of 32,162 values (i.e., 0.24%) were outside of this range. Where appropriate, these values have been recoded to their closest minimum or maximum value (i.e., |3|).

Table SI1. *Descriptive Statistics on Raw Scores for Full Sample.*

| Variable | N | Mean | SD | Median | Min | Max | Range | Skew | Kurtosis |
| --- | --- | --- | --- | --- | --- | --- | --- | --- | --- |
| Verbal Fluency | 2681 | 17.31 | 4.47 | 17.33 | 3.67 | 37.33 | 33.67 | 0.19 | 0.43 |
| VEM (free recall) | 2638 | 8.48 | 3.96 | 8.00 | 1.00 | 20.00 | 19.00 | 0.82 | 0.41 |
| VEM (recognition) | 2642 | 0.65 | 0.20 | 0.64 | -0.04 | 1.00 | 1.04 | -0.32 | -0.41 |
| Face Recognition | 2649 | 0.39 | 0.19 | 0.40 | -0.35 | 0.95 | 1.30 | -0.18 | -0.17 |
| Emotion Recognition | 2604 | 27.60 | 3.57 | 28.00 | 10.00 | 36.00 | 26.00 | -0.61 | 0.66 |
| Line Angle Judgment | 2754 | 10.02 | 4.19 | 10.00 | 0.00 | 20.00 | 20.00 | 0.28 | -0.67 |
| Mental Rotation | 2612 | 14.96 | 2.61 | 15.00 | 6.00 | 20.00 | 14.00 | -0.41 | -0.20 |
| Openness | 2610 | 3.77 | 0.60 | 3.83 | 1.67 | 5.00 | 3.33 | -0.34 | -0.23 |
| Conscientiousness | 2610 | 3.64 | 0.61 | 3.67 | 1.42 | 5.00 | 3.58 | -0.31 | -0.18 |
| Extraversion | 2610 | 3.29 | 0.63 | 3.33 | 1.08 | 4.92 | 3.83 | -0.13 | -0.31 |
| Agreeableness | 2610 | 3.86 | 0.50 | 3.92 | 1.83 | 5.00 | 3.17 | -0.48 | 0.22 |
| Neuroticism | 2610 | 2.71 | 0.71 | 2.67 | 1.00 | 4.83 | 3.83 | 0.24 | -0.36 |
| Interest in People | 2741 | 3.82 | 0.74 | 3.88 | 1.25 | 6.00 | 4.75 | -0.27 | 0.13 |
| Interest in Things | 2741 | 3.01 | 1.22 | 3.00 | 1.00 | 6.00 | 5.00 | 0.20 | -0.87 |

*Note.* VEM = Verbal Episodic Memory.

Table SI2. *Extreme Scores.*

|  | Below 10th percentile | | |  | Above 90th percentile | | |
| --- | --- | --- | --- | --- | --- | --- | --- |
| Variable | Male N | Female N | Ratio |  | Male N | Female N | Ratio |
| Verbal Fluency | 146 | 97 | 1.51 |  | 85 | 164 | 0.52 |
| Verbal Episodic Memory | 169 | 87 | 1.94 |  | 81 | 172 | 0.47 |
| Face Recognition | 140 | 106 | 1.32 |  | 40 | 126 | 0.32 |
| Emotion Recognition | 130 | 101 | 1.29 |  | 66 | 88 | 0.75 |
| Line Angle Judgment | 57 | 166 | 0.34 |  | 173 | 53 | 3.26 |
| Mental Rotation | 24 | 115 | 0.21 |  | 150 | 36 | 4.17 |
| Openness | 113 | 115 | 0.98 |  | 111 | 150 | 0.74 |
| Conscientiousness | 165 | 82 | 2.01 |  | 50 | 158 | 0.32 |
| Extraversion | 157 | 104 | 1.51 |  | 96 | 158 | 0.61 |
| Agreeableness | 137 | 73 | 1.88 |  | 73 | 133 | 0.55 |
| Neuroticism | 174 | 66 | 2.64 |  | 84 | 162 | 0.52 |
| Interest in People | 160 | 85 | 1.88 |  | 59 | 151 | 0.39 |
| Interest in Things | 26 | 194 | 0.13 |  | 227 | 39 | 5.82 |

Note. Extreme scores defined as scores above the 90th (top) or below the 10th percentile (bottom) of the combined sample distribution. Ratios represent the number of males relative to females at each threshold. Ratios > 1 indicate male overrepresentation; ratios < 1 indicate female overrepresentation.

# Logistic Regression

In the main manuscript, we report logistic regressions in four steps, but do not present each variable in simple models. These models are described in Table SI3, below.

Table SI3. *Logistic Regression Coefficients with 95% Confidence Intervals for Each Study Variable.*

| Model | 95 % LL | OR | 95 % UL | *p* | Tjur's R2 | McFadden’s R2 |
| --- | --- | --- | --- | --- | --- | --- |
| Verbal Fluency | 1.24 | 1.35 | 1.47 | 0.00 | 0.02 | 0.02 |
| Verbal Episodic Memory | 1.41 | 1.54 | 1.67 | 0.00 | 0.04 | 0.03 |
| Face Recognition | 1.24 | 1.34 | 1.46 | 0.00 | 0.02 | 0.02 |
| Emotion Recognition | 1.13 | 1.22 | 1.32 | 0.00 | 0.01 | 0.01 |
| Line Angle Judgment | 0.44 | 0.48 | 0.53 | 0.00 | 0.11 | 0.08 |
| Mental Rotation | 0.42 | 0.46 | 0.50 | 0.00 | 0.12 | 0.09 |
| Openness | 1.03 | 1.11 | 1.21 | 0.01 | 0.00 | 0.00 |
| Conscientiousness | 1.42 | 1.54 | 1.68 | 0.00 | 0.04 | 0.03 |
| Extraversion | 1.28 | 1.39 | 1.51 | 0.00 | 0.03 | 0.02 |
| Agreeableness | 1.31 | 1.42 | 1.55 | 0.00 | 0.03 | 0.02 |
| Neuroticism | 1.45 | 1.58 | 1.72 | 0.00 | 0.05 | 0.04 |
| Interest in People | 1.36 | 1.48 | 1.60 | 0.00 | 0.04 | 0.03 |
| Interest in Things | 0.28 | 0.31 | 0.35 | 0.00 | 0.23 | 0.18 |

*Note.* These coefficients are computed from simple logistic regressions, meaning that this is 13 separate regression models. All *p*-values are < .0001 except for Openness for which *p* = .008.

# Dominance Analysis

To address concerns about the sensitivity of results to predictor entry order, we conducted dominance analysis (Azen & Budescu, 2003) using the *dominanceanalysis* package in R (Bustos & Soares, 2020). Dominance analysis evaluates the relative importance of predictors by comparing their additional contribution to model fit (McFadden's pseudo-R²) across all possible sub-models. For *k* predictors, this involves fitting all 2^*k* subset models. We report general dominance weights, defined as each predictor's average incremental contribution to R² across all possible subsets of the remaining predictors. These weights are order-invariant and sum to the full model R². We conducted the analysis in two ways: at the individual predictor level (13 predictors, 8,192 sub-models) and at the block level, grouping predictors into cognitive abilities (verbal fluency, verbal episodic memory, face recognition, emotion recognition, line angle judgment, mental rotation), personality traits (Big Five), and interests (people-orientation, things-orientation). Figure S1 displays weights on the block level and Figure S2 displays weights on the individual variable level.

The full model (i.e., Model 4) McFadden's pseudo-R² was .376. At the individual predictor level, interest in things was the strongest single predictor, contributing an average of .117 to model R² across all possible sub-models. Mental rotation (.054) and line angle judgment (.062) were the next strongest contributors, followed by neuroticism (.038) and verbal episodic memory (.029). The remaining predictors each contributed less than .024. Interest in things achieved complete dominance over all other individual predictors.^^[[1]](#footnote-1)^^

At the block level, cognitive abilities collectively contributed the most to model fit (.168), followed by interests (.125) and personality (.087). Complete dominance held across all pairwise comparisons: cognitive abilities dominated both interests and personality, and interests dominated personality. This ordering was consistent across all four fit indices examined (McFadden, Cox-Snell, Nagelkerke, and Estrella pseudo-R²). Critically, because dominance analysis evaluates predictor importance across all possible orderings, these results are not contingent on the order in which variables are entered into the model. The results for both variants are plotted below.

# Cross-validation

To evaluate the generalizability of Model 4, we conducted 10-fold cross-validation using the caret package in R (Kuhn, 2008). The dataset was randomly partitioned into 10 equally sized folds. In each iteration, nine folds were used to fit the logistic regression model and the held-out fold was used to generate predictions. Model discrimination was evaluated using area under the receiver operating characteristic curve (AUC), accuracy males (sensitivity), and accuracy females (specificity). Cross-validated performance (AUC = .878, classification accuracy for males = .763, classification accuracy for females = .820) was near-identical to the full-sample estimates (AUC = .878 [95% CI: .864, .898], accuracy for males = .770, accuracy for females = .823), indicating minimal overfitting. Across the 10 folds, AUC ranged from .867 to .890, accuracy for males from .722 to .817, and accuracy for females from .803 to .886.


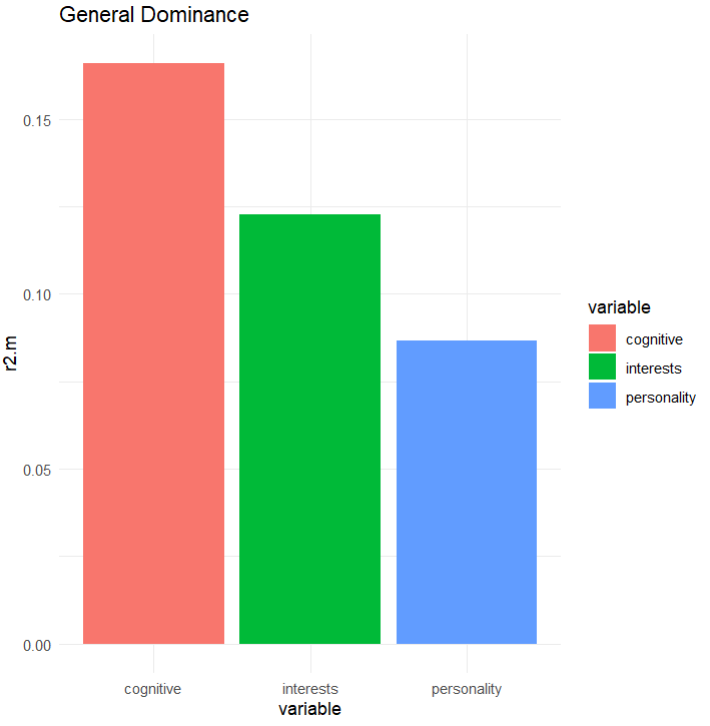


*Figure SI1****.*** General dominance weights for predictor blocks in the sex classification. Bars represent each block's average incremental contribution to McFadden's pseudo-R² across all eight possible sub-models. Cognitive abilities comprised verbal fluency, verbal episodic memory, face recognition, emotion recognition, line angle judgment, and mental rotation; personality comprised the Big Five traits; interests comprised people-orientation and things-orientation. Complete dominance held for all pairwise comparisons (cognitive > interests > personality).


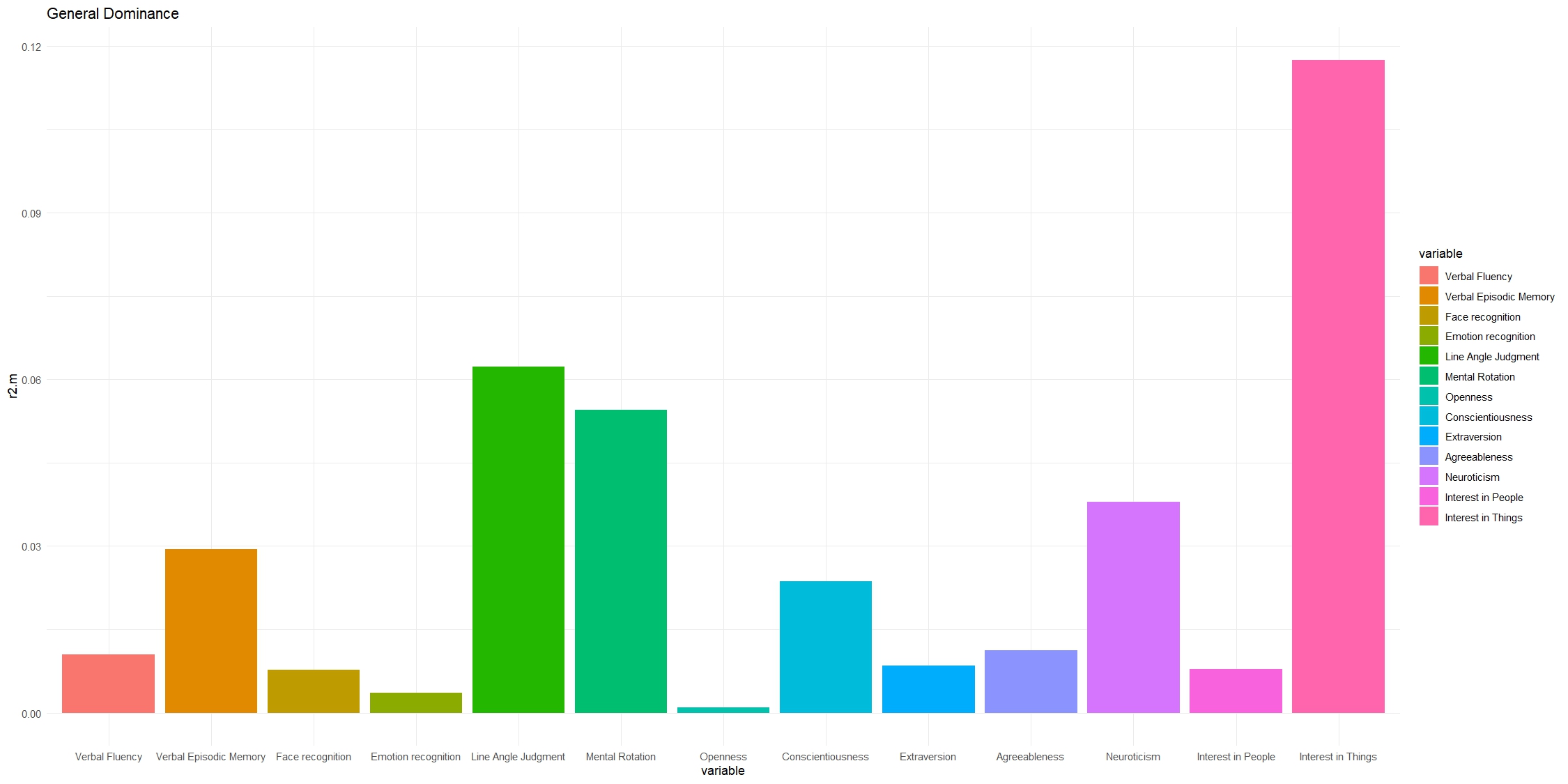


*Figure SI2.* General dominance weights for individual predictors of sex classification. Bars represent each predictor's average incremental contribution to McFadden's pseudo-R² across all 8,192 possible sub-models. General dominance weights sum to the full model R² (.376). Interest in things was the strongest single predictor and achieved complete dominance over all 12 remaining predictors.

# Beta Regression

Here, we report coefficients from all three beta regressions using gender segregation in occupations as criterion. For all three models, both average marginal effects and ordinary beta regression coefficients are reported. Model 1 is simply using only sex as a predictor. Model 2 uses all tasks and tests without controlling for sex, and Model 3 combines these two models.

As an example for how to interpret these models, interest in things was the strongest predictor of gender segregation in occupation. In Model 2 (all individual differences, no sex), the coefficient was β = -0.35, AVE = -0.083, *p* < .001, indicating that a one standard deviation increase in interest in things was associated with an 8.3 percentage point shift toward more male-typed occupations, on average. When sex was added in Model 3, the coefficient attenuated to β = -0.31, AVE = -0.072, *p* < .001, suggesting that part of the association between interest in things and occupational gender segregation is shared with sex, but a substantial independent contribution remains. This is consistent with the dominance analysis, which identified interest in things as the single most important predictor of sex.

Table SI4

*Regression Results Using Gender Segregation in Occupation as Criterion*

|  | Model 1 | | |  | Model 2 | | |  | Model 3 | | |
| --- | --- | --- | --- | --- | --- | --- | --- | --- | --- | --- | --- |
| Predictor | Beta | *p* | *AVE* |  | Beta | *p* | *AVE* |  | Beta | *p* | *AVE* |
| Intercept | -0.26 | < .001 |  |  | 0.12 | < .001 |  |  | -0.06 | 0.05 |  |
| Sex | 0.72 | < .001 | 0.177 |  |  |  |  |  | 0.34 | < .001 | 0.083 |
| Verbal Fluency |  |  |  |  | 0.03 | 0.18 | 0.006 |  | 0.01 | 0.50 | 0.003 |
| Verbal Episodic Memory |  |  |  |  | 0.05 | 0.01 | 0.011 |  | 0.02 | 0.33 | 0.004 |
| Face Recognition |  |  |  |  | 0.01 | 0.71 | 0.002 |  | 0.00 | 0.93 | 0.000 |
| Emotion Recognition |  |  |  |  | 0.00 | 0.97 | 0.000 |  | -0.01 | 0.73 | -0.002 |
| Line Angle Judgment |  |  |  |  | -0.07 | < .001 | -0.017 |  | -0.03 | 0.08 | -0.008 |
| Mental Rotation |  |  |  |  | -0.09 | < .001 | -0.022 |  | -0.07 | 0.001 | -0.016 |
| Openness |  |  |  |  | 0.02 | 0.21 | 0.006 |  | 0.03 | 0.19 | 0.006 |
| Conscientiousness |  |  |  |  | 0.02 | 0.37 | 0.004 |  | -0.01 | 0.79 | -0.001 |
| Extraversion |  |  |  |  | -0.06 | 0.01 | -0.015 |  | -0.07 | < .001 | -0.017 |
| Agreeableness |  |  |  |  | 0.03 | 0.15 | 0.007 |  | 0.02 | 0.47 | 0.004 |
| Neuroticism |  |  |  |  | 0.04 | 0.08 | 0.009 |  | 0.00 | 0.96 | 0.000 |
| Interest in People |  |  |  |  | 0.10 | < .001 | 0.023 |  | 0.1 | < .001 | 0.023 |
| Interest in Things |  |  |  |  | -0.35 | < .001 | -0.083 |  | -0.31 | < .001 | -0.072 |
| Phi coefficient (Φ) | 3.58 | < .001 |  |  | 3.95 | < .001 |  |  | 4.04 | < .001 |  |

*Note.* Beta = beta regression coefficient on the logit-link scale. AVE = average marginal effect, representing the average change in the outcome on its original (0, 1) scale for a one-unit increase in the predictor. *p* = *p*-value for the beta regression coefficient. Φ = precision parameter of the beta regression model, where larger values indicate less dispersion in the outcome variable around the predicted mean.

# Additional figures

In Figure SI4, the distribution of z-scores divided by sex for the study variables can be seen. This figure essentially displays the same information as Table 1 in the main manuscript. Figure SI5 shows predicted probabilities from the 13 simple regression models, so essentially the same concept as Figure 1 in the main manuscript, but this is for each simple model and not the probabilities that result from using multiple predictors simultaneously. Finally, Figure SI6 shows ROC-curves for each of the four main models, where incremental improvement in sensitivity and specificity is highlighted.


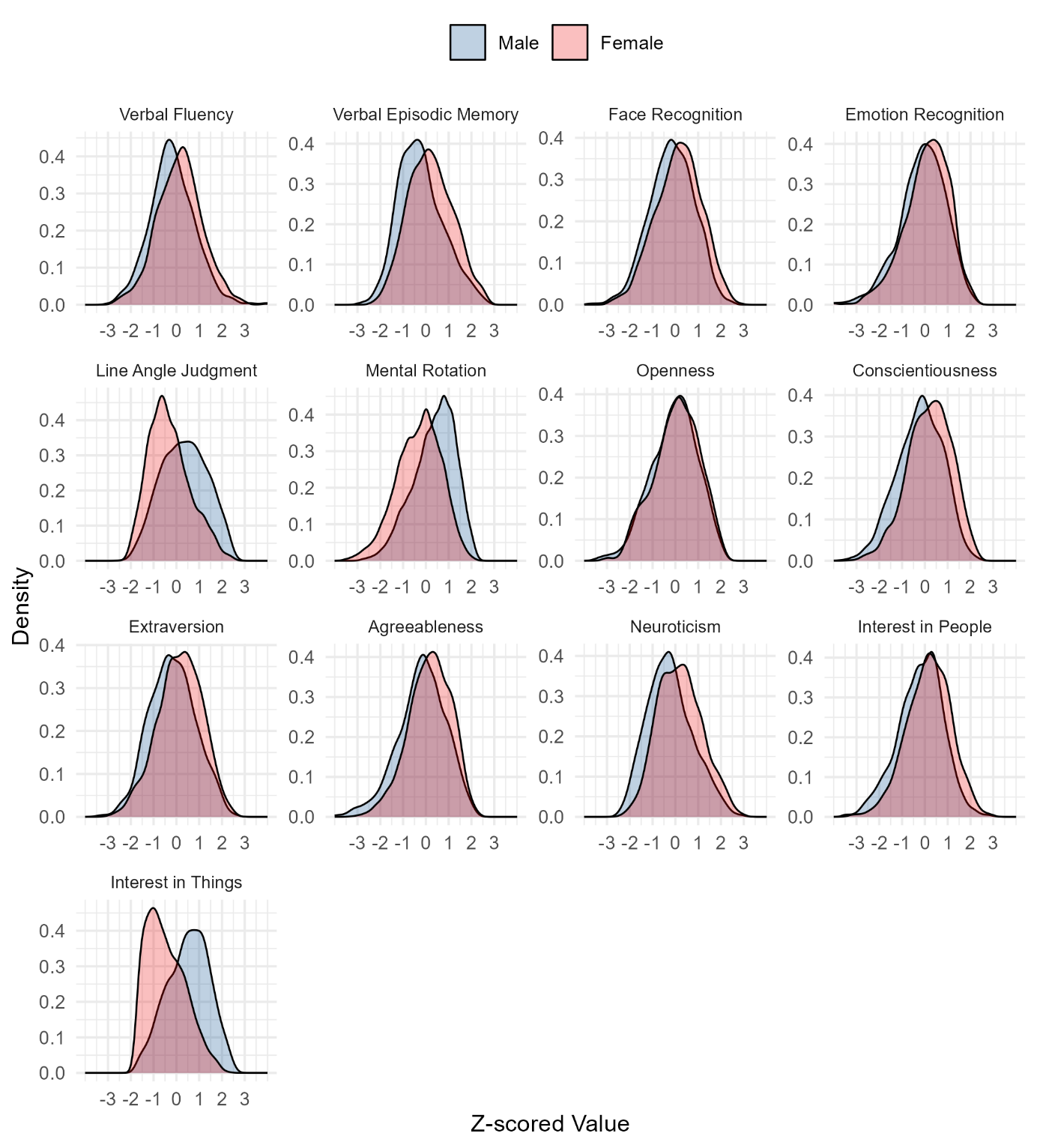


*Figure SI3*. Distributions of sex differences in z-scores for all study variables. A total of 79 values (from a total of 34,470) that fall outside the x-axis scope of |3| have been recoded to their closest minimum or maximum value (i.e., |3|).


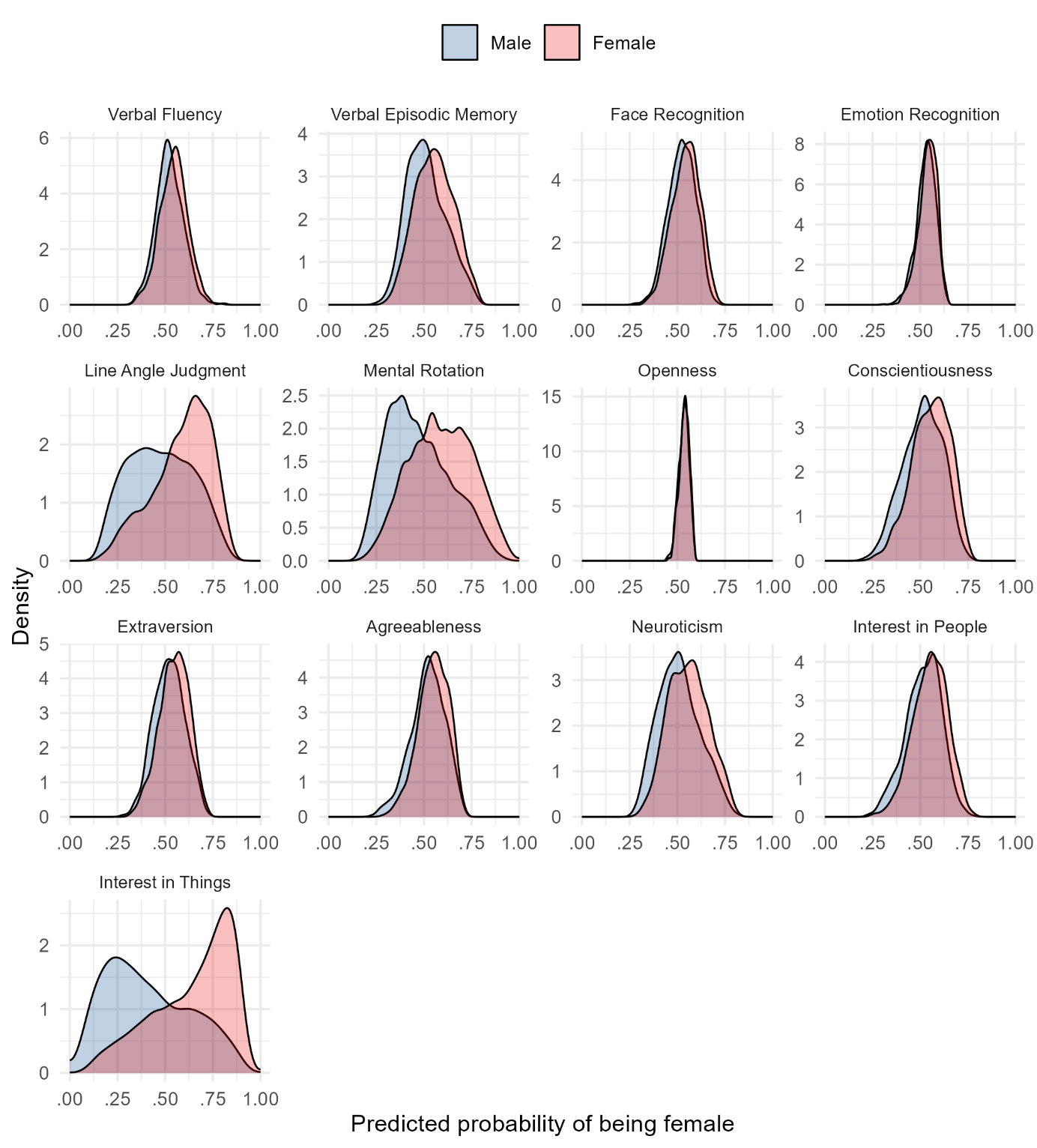


*Figure SI4.* Predicted probabilities from each individual predictor. Note that females are coded as 1 and males as 0.


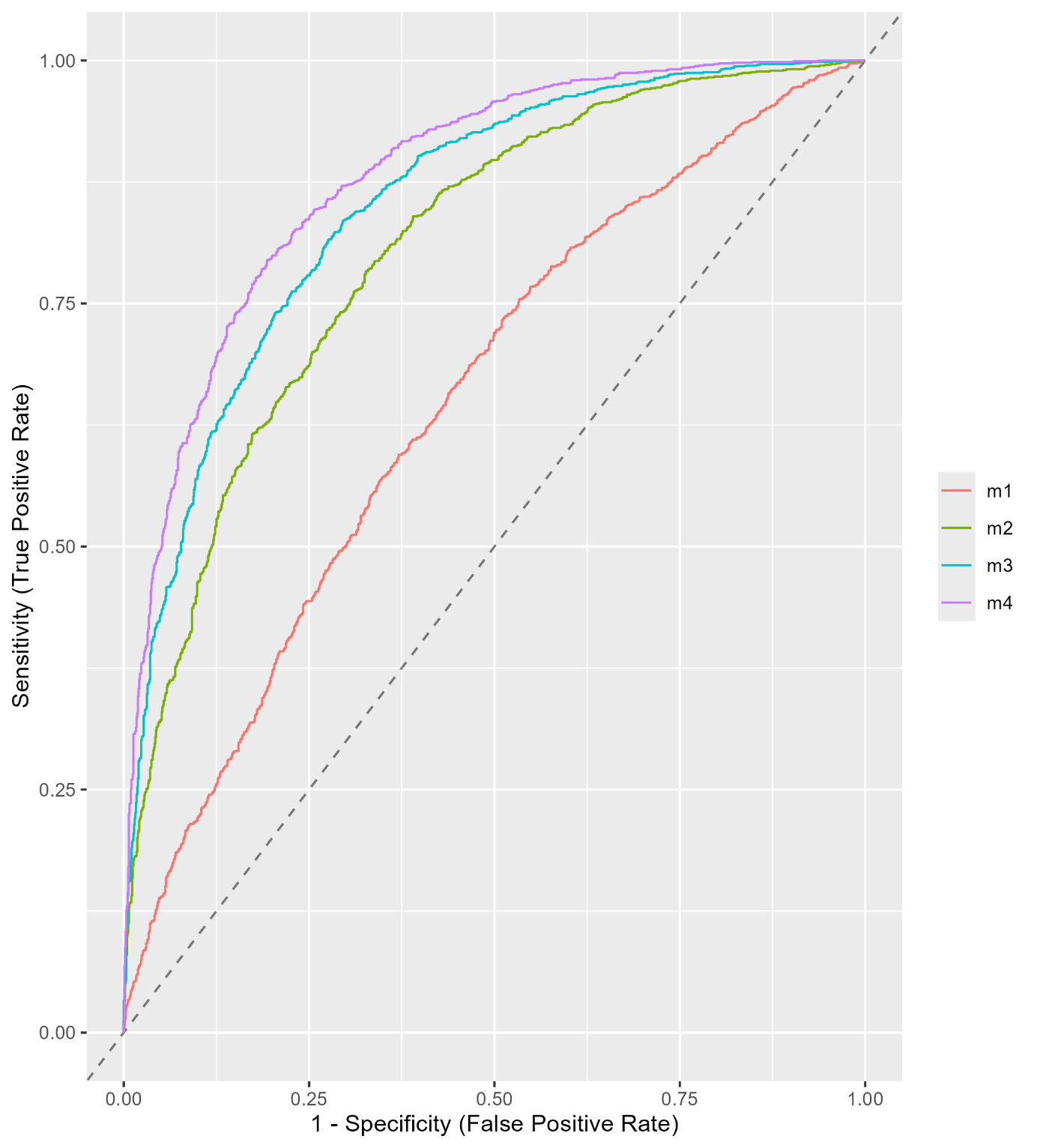


*Figure SI5*. ROC graph showing incremental improvements for each model (m1-m4).

References

Azen, R., & Budescu, D. V. (2003). The dominance analysis approach for comparing predictors in multiple regression. *Psychological methods*, *8*(2), 129-148. <https://doi.org/10.1037/1082-989x.8.2.129>

Kuhn, M. (2008). Building Predictive Models in R Using the caret Package. Journal of Statistical Software, 28(5), 1–26. <https://doi.org/10.18637/jss.v028.i05>

Navarrete, C. B., Soares, F. C., & Navarrete, M. C. B. (2020). *Package ‘dominanceanalysis’*. <https://cran.r-project.org/web/packages/dominanceanalysis/index.html>

1. Predictor A completely dominates predictor B if A's incremental R² contribution is greater than B's incremental R² contribution in every possible subset of the other predictors, including the empty model (just A vs. just B) and the model with all other predictors included. So for interest in things to completely dominate mental rotation, for instance, it means: for every possible combination of the remaining 11 predictors, adding interest in things improved fit more than adding mental rotation. Across all 2^11 = 2,048 such comparisons, interest in things always won. And this held for all 12 pairwise comparisons. [↑](#footnote-ref-1)
